# *Chromobacterium violaceum*: Important Insights for Virulence and Biotechnological Potential by Exoproteomic Studies

**DOI:** 10.1007/s00284-013-0334-5

**Published:** 2013-02-28

**Authors:** Alessandra Ciprandi, Wanderson Marques da Silva, Agenor Valadares Santos, Adriano Monteiro de Castro Pimenta, Marta Sofia Peixe Carepo, Maria Paula Cruz Schneider, Vasco Azevedo, Artur Silva

**Affiliations:** 1 Núcleo de Medicina Tropical, Universidade Federal do Pará, Av. Gen. Deodoro 92, Belém, 66055-240 Brazil; 2 Laboratório de Polimorfismo de DNA, Instituto de Ciências Biológicas, Universidade Federal do Pará, Rua Augusto Corrêa s/n, Belém, 66075-900 Brazil; 3 Departamento de Biologia Geral, Instituto de Ciências Biológicas, Universidade Federal de Minas Gerais, Av. Antônio Carlos 6627, Belo Horizonte, 31270-910 Brazil; 4 Departamento de Bioquímica e Imunologia, Instituto de Ciências Biológicas, Universidade Federal de Minas Gerais, Av. Antônio Carlos 6627, Belo Horizonte, 31270-901 Brazil; 5 REQUIMTE/CQFB, Departamento de Química, Faculdade de Ciências e Tecnologia, Universidade Nova de Lisboa, 2829-516 Caparica, Portugal

## Abstract

*Chromobacterium violaceum* is a beta-proteobacterium with high biotechnological potential, found in tropical environments. This bacterium causes opportunistic infections in both humans and animals, that can spread throughout several tissues, quickly leading to the death of the host. Genomic studies identified potential mechanisms of pathogenicity but no further studies were done to confirm the expression of these systems. In this study 36 unique protein entries were identified in databank from a two-dimensional profile of *C. violaceum* secreted proteins. *Chromobacterium violaceum* exoproteomic preliminary studies confirmed the production of proteins identified as virulence factors (such as a collagenase, flagellum proteins, metallopeptidases, and toxins), allowing us to better understand its pathogenicity mechanisms. Biotechnologically interesting proteins (such as chitinase and chitosanase) were also identified among the secreted proteins, as well as proteins involved in the transport and capture of amino acids, carbohydrates, and oxidative stress protection. Overall, the secreted proteins identified provide us important insights on pathogenicity mechanisms, biotechnological potential, and environment adaptation of *C. violaceum.*

## Introduction

*Chromobacterium violaceum* is a gram-negative β-proteobacterium commonly found in the soil and water of tropical and sub-tropical regions. Like other free-living microbes, its metabolism is characterized by its versatility, which enables the bacterium to adapt to the diverse environmental conditions to which it is exposed [6]. This bacterium can also potentially produce several useful compounds for environmental detoxification, bioprospecting, pest control, and therapeutics. Violacein, a pigment that displays cytotoxic and antibacterial activity, is an example of such a compound [8, 18].

*Chromobacterium violaceum* is an opportunistic pathogen for both animals and humans, with cases reported in Southeast Asia, Oceania, and the Americas [14]. The dominant route of infection for this pathogen is through exposure of injured skin to contaminated water or soil, with effects ranging from cutaneous lesions and visceral abscesses to severe sepsis, which progresses rapidly to death [17]. The quick evolution of disease and the antibiotic treatment failure result in a mortality rate of over 60 % [14].

The analysis of *C. violaceum* genome identified several putative virulence factors, of which none have been characterized at the molecular level. Among these candidates are type II and type III secretion systems, cytolytic toxins (hemolysins and leukotoxins), metalloproteases, and lipases [7].

Protein secretion is one of the most important means by which bacteria interact with their environment. The proteins that are released into the extracellular medium have a wide range of functions, including nutrient acquisition, stress protection, and the development of host-microbe associations via the formation of biofilms for cellular adhesion and host colonization [37, 38]. This study aims to identify in the exoproteome of *C. violaceum* proteins that provide us insights on its pathogenicity mechanisms, interactions between the bacteria and their environment, stress protection, and biotechnological potential.

## Materials and Methods

### Bacterial Strains and Growth Conditions

For the isolation of the extracellular proteins, *C. violaceum* ATCC 12472 was grown in 1 L of a chemically defined medium [1.29 % Na_2_HPO_4_, 0.25 % KH_2_PO_4_, 0.1 % NH_4_Cl, 0.002 % CaCl_2_, 0.02 % MgSO_4_, 2.4 % glucose, 0.05 % Tween-80, 4 % vitamin solution (MEM vitamin solution, Invitrogen), 1 % essential amino acids solution (MEM essential amino acids, Invitrogen), and 1 % non-essential amino acids solution (MEM non-essential amino acids, Invitrogen)] at 28ºC in a rotating shaker (140 rpm) until the mid-exponential growth phase (OD_720_ = 0.8) [16].

### Extraction of Extracellular Proteins

The culture medium was centrifuged at 4,000×*g* for 20 min at 4ºC. The supernatant was collected and filtered through a 0.22-μm pore-diameter membrane. The proteins were extracted using a three-phase partitioning method [33]. Ammonium sulfate was added to the clarified supernatant to a final concentration of 30 %, and the pH was adjusted to 4.0. Subsequently, *n*-butanol was added in a 1:1 ratio to the filtered supernatant, and the mixture was incubated for 1 h at room temperature. Phase separation occurred after centrifugation at 2,000×*g* for 10 min. The interfacial precipitate was collected and resuspended in 20 mM Tris–HCl pH 7.4 supplemented with a protease inhibitor cocktail (GE Healthcare). The suspension was dialyzed for 48 h against Milli-Q-purified water using a dialysis membrane with a 12-kDa cut-off (Sigma). The protein concentration was determined using the Bradford method [5]. Three separate protein extractions were performed from each of three independently grown cultures of *C. violaceum*.

### Two-Dimensional Gel Electrophoresis (2DE)

The extracellular proteins (180 μg) were precipitated using methanol/chloroform and dissolved in a rehydration solution (7 M urea, 2 M thiourea, 2 % CHAPS, 1 % pH 3–11 NL ampholytes, 75 mM DTT, and 0.002 % bromophenol blue). The same solution was used to rehydrate the gel strips for isoelectric focusing (IEF) (pH 3–11 NL, 18 cm, GE Healthcare). IEF was performed on an Ettan™ IPGphor™ (GE Healthcare) apparatus until 80,000 Vh. SDS-PAGE was performed on the DALTsix (GE Healthcare) vertical apparatus using a homogeneous 15 % polyacrylamide gel. Proteins were stained with Colloidal Coomassie blue [31]. Three biological replicates of 2-DE gels were digitized with an ImageScanner™ (GE Healthcare), and the resulting images were analyzed using the ImageMaster™ 2D Platinum v7.0 software (GE Healthcare).

### Tryptic Digestion and Mass Spectrometry

All spots from 2DE were picked from the gel using an Ettan™ Spot Picker (GE Healthcare). The tryptic digestion was performed according to Havlis et al. [24]. The peptides were concentrated, desalted using a C18 ZipTip^®^ (Millipore, Bellerica, MA), and stored at −20ºC.

Aliquots (0.5 μL) of the peptide solutions were mixed with 0.5 μL of a 10 mg/mL α-cyano-4-hydroxycinnamic acid matrix, spotted onto an AnchorChip™ 600/384 (Bruker Daltonics, Bremen, Germany) target microtiter plate (MTP), and analyzed with a MALDI-TOF/TOF AutoFlex III mass spectrometer (Bruker Daltonics). The results of the MS/MS analysis were used to search the NCBI protein database using the MASCOT^®^ software. The search parameters were as follows: type of search, peptide mass fingerprint combined with MS/MS ion search; amino acid sequence, enzyme, trypsin; fixed modification, carbamidomethylation (Cys); variable modifications, oxidation (Met); mass values, monoisotopic; peptide charge state, 11; maximum missed cleavages, 1; and a peptide mass tolerance of 0.05 % Da (50 ppm).

### Bioinformatics Tools

The prediction of *C. violaceum* protein subcellular localization was performed using the SurfG+ software [2]. SecretomeP 2.0, a software available online at http://www.cbs.dtu.dk/services/SecretomeP/, was used to evaluate secretion via the non-classical pathway. The COG database (http://www.ncbi.nlm.nih.gov/COG/) was used to obtain a functional classification of the proteins. The protein sequence comparisons were performed using BLAST (http://www.ncbi.nlm.nih.gov/BLAST/).

### Results and Discussion

*Chromobacterium violaceum* was grown in a chemically defined medium, and the extracellular proteins were obtained using a three-phase fractionation method [33].

**Fig. 1:**
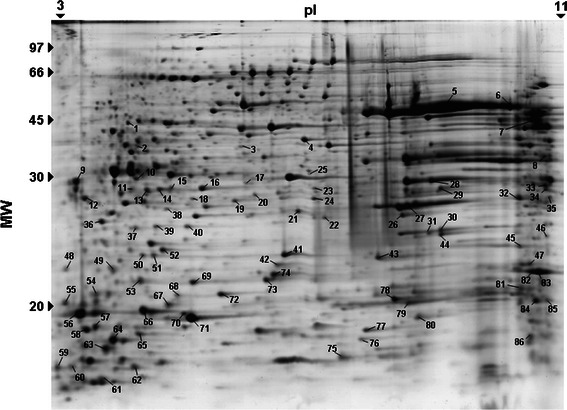
Two-dimensional map of the secreted proteins of *C. violaceum*, which are stained with colloidal Coomassie blue. The spot numbers refer to Table 1

The secreted proteins were separated using 2DE, which resulted in a profile containing 338 spots (Fig. 1). All spots were selected and subjected to mass spectrometry analysis. Of these spots, 86 were identified as 36 protein entries by MS/MS followed by databank searching (Table 1). Several spots with different pI and MM values corresponded to the same protein entry, which was likely due to posttranslational modifications, such as the addition of prosthetic groups and/or proteolytic processing. Similar results have been reported in the literature for other exoproteome analyses, such as in *Streptococcus suis* [39], *Herbaspirillum seropedicae* [9], *Rhodococcus equi* [1], and *Corynebacterium pseudotuberculosis* [32].

**Table 1:** Extracellular proteins of *C. violaceum* identified using MALDI-TOF/TOF MS

Spot nº	Locus tag	Protein	Cellular localization^a^	COG Class^b^
1, 28, 32, 40, 51, 64, 67, 71	CV_1097	Binding protein component of ABC dipeptide transporter	E	E
2	CV_1709	Flagellar hook-associated protein FlgK	C	N
3	CV_3931	Chitosanase A	E	G
4, 10, 33, 43, 44, 82	CV_2001	Collagenase	C	O
5	CV_3475	Hypothetical protein CV_3475	E	S
6, 8, 18, 35, 45, 46, 52, 53, 57, 59, 70, 76, 77, 79	CV_1440	Hydrolase transmembrane protein	E	R
7	CV_4329	Oligopeptide ABC transporter system, substrate-binding protein	E	E
9, 16	CV_3739	Peroxidase	C	P
11, 85	CV_0349	Hypothetical protein CV_0349	C	S
12, 42, 58, 60, 63, 68, 69, 86	CV_4107	Hypothetical protein CV_4107	PSE	S
13, 19, 21	CV_1710	Flagellar hook-associated protein FlgL	C	N
14	CV_4224	Hypothetical protein CV_4224	C	S
15, 27, 31, 41	CV_0350	Phage sheath protein	C	R
17, 18, 66	CV_3323	Carbohydrate-binding protein	E	S
20, 49, 56	CV_2994	Flagellar hook-associated protein FliD	C	N
22, 36	CV_1706	Flagellar basal body rod protein FlgG	C	N
23, 24, 55, 62, 78	CV_3977	Hypothetical protein CV_3977	C	S
25	CV_0362	Thermolabile hemolysin, lecithin-dependent	C	R
26	CV_0867	Superoxide dismutase	E	P
29, 30	CV_1369	Hypothetical protein CV_1369	E	S
34	CV_3276	Hypothetical protein CV_3276	E	S
36, 54, 61	CV_2893	Hypothetical protein CV_2893	E	S
37, 83	CV_4188	Elongation factor Tu	C	J
38, 39, 47, 49, 73, 75	CV_3011	Flagellin D	C	N
48	CV_0223	Hypothetical protein CV_0223	C	H
50	CV_3506	Protease precursor	E	R
54	CV_0408	Hypothetical protein CV_0408	C	S
57	CV_1703	Flagellar basal body rod modification protein	C	N
65	CV_4240	Chitinase	E	G
71	CV_2390	Riboflavin synthase subunit beta	C	H
71	CV_0340	Tail fiber assembly protein	C	R
72	CV_0424	Hypothetical protein CV_0424	C	R
74	CV_1415	γ-glutamyltransferase precursor	E	E
80	CV_0410	Bacteriophage tail core protein	C	R
81	CV_2034	Peptidoglycan *N*-acetylmuramoyl hydrolase	E	M
84	CV_0409	Bacteriophage tail sheath protein	C	R

^a^ *E* extracellular protein, *C* cytoplasmic protein, *PSE* potentially surface-exposed protein, according to the SurfG prediction

^b^Classes of clusters of orthologous groups (COG): (E) amino acid transport and metabolism; (G) carbohydrate transport and metabolism; (H) coenzyme metabolism; (J) translation, ribosomal structure, and biogenesis; (M) cell envelope biogenesis; (N) cell motility and secretion; (O) posttranslational modification, protein turnover, chaperones; (P) inorganic ion transport and metabolism; (R) general function prediction only; (S) function unknown

Of the identified protein entries, SurfG+ only predicted that 14 were localized extracellularly, 21 were cytoplasmic, and one was exposed on the cell surface (Table 1). To predict non-classical pathway secretion, the 21 proteins predicted by SurfG+ to be cytoplasmic were submitted to the SecretomeP program. Thirteen proteins exhibited SecP scores higher than 0.5, consistent with non-classical export pathways. In silico *C. violaceum* genome analysis with SurfG+ predicted 433 extracellular proteins, ~10 % of all of the *C. violaceum* ORFs.

Eight protein entries (25 %) were predicted to be cytoplasmic using all of the prediction methods. Some of those proteins may have been released into the extracellular medium due to cellular lysis, and others via some unknown mechanism to perform a different function such as moonlighting proteins [26]. The elongation factor Tu (EF-Tu) CV_4188 is a moonlighting protein found in exoproteomes [1, 9, 29]; different from its function as a translation factor, this protein can be combined with the membrane and localized to the cell surface to perform a new function related to pathogenicity. In *Mycoplasma pneumoniae*, EF-Tu can bind to fibronectin, an adhesion glycoprotein of the extracellular matrix [12, 40]. In *Pseudomonas aeruginosa*, the cell surface-bound EF-Tu can serve as a receptor for host factor H proteins and plasminogen, which allows the bacterium to evade the immune system and invade the host [27]. The presence of the EF-Tu CV_4188 in the *C. violaceum* exoproteome suggests the involvement of this protein in cell adhesion mechanisms.

Among the identified exoproteins, many are involved in cellular motility, all of which belong to the flagellar apparatus (CV_1703, CV_1706, CV_1709, CV_1710, CV_2994, and CV_3011). The flagellum may contribute to pathogenicity as a non-flagellar protein secretion system, and this apparatus may possess additional functions such as cell adhesion [28].

In agreement with the disseminated infections caused by *C. violaceum*, several other virulence factors were identified including a collagenase (CV_2001), which may be involved in tissue necrosis and cytopathic effects [23], and a lecithin-dependent thermolabile hemolysin (CV_0362). Some strains of *C. violaceum* exhibit hemolytic activity, and 13 ORFs related to the hemolysins are present in the *C. violaceum* ATCC 12472 genome [7]. The hemolysin identified in this study show 40 % sequence identity with those of *Vibrio parahaemolyticus* [35] and *Legionella pneumophila* [20], which possess phospholipase A activity and are cytolytic toxins. Protein CV_3977 corresponds to a hemolysin-coregulated protein that belongs to the type VI secretion system. The extracellular, zinc-dependent metallopeptidase CV_3506 and the protein CV_4107, both entries identified in this study, are similar to known virulence factors commonly produced by bacteria [22, 29]. However, none of the *C. violaceum* type III secretion system effector proteins that were predicted by Betts et al. [3] were identified; this may be because their expression can only be activated when in contact with a host cell.

Metabolic enzymes can also play a role in the virulence of *C. violaceum*, such as gamma-glutamyltransferase (CV_1415). This periplasmic enzyme has a role in cysteine recycling and glutathione metabolism, and it serves as a virulence factor in *Helicobacter pylori*, inducing apoptosis and modulating inflammation [4]. Riboflavin synthase, such as CV_2390, was also described as a virulence factor in *Salmonella enterica, Mycobacterium leprae* [19], and *H. pylori* [11] by providing riboflavin to extracellular ferric reductases, which have a role in increasing iron bioavailability. Peptidoglycan *N*-acetylmuramoyl hydrolase CV_2034 may be important in the cell wall turnover process, but it can also act as defense mechanism against other bacteria [25] or as a virulence factor [41].

**Fig. 2:**
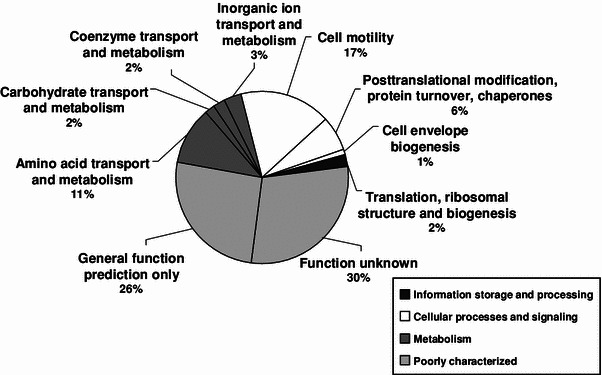
Functional classification of *C. violaceum* extracellular proteins

The functional classification of the *C. violaceum* extracellular proteins revealed that over 50 % belonged to the poorly characterized category, and 25 % were linked to metabolic or transport roles, while a single protein was found to be involved in information processing (Fig. 2). Two substrate-binding proteins were found in the exoproteome: one binding protein that is part of the ATP-binding cassette (ABC) oligopeptide transport system, CV_4329, and an additional binding protein that belongs to the ABC dipeptide transport system, CV_1097. Both have a role in capturing oligopeptides that can serve as sources of amino acids for the cell. Oligopeptide-binding proteins can also act as intracellular signals, take part in adhesion processes, and serve as molecular chaperones [30].

Several protein entries that might be involved in carbohydrate metabolism were identified, among them are a chitosanase (CV_3931) and a chitinase (CV_4240). This finding is the first reported evidence of a chitosanase in *C. violaceum*, and our results also indicated that this enzyme is likely to be expressed constitutively, unlike most bacterial chitosanases, which are inducible. Chitosanase is responsible for the breakdown of chitosan, producing chitooligosaccharides with potential anti-tumor and anti-bacterial activities that are of great interest in both the pharmaceutical and food industries [34].

Likewise, chitinase was also expressed without chitin induction, which presents potential applications in the biological control of insects and fungi. Chernin et al. [10] detected six different types of chitinolytic activity in *C. violaceum* using a synthetic substrate, all dependent on chitin induction and the quorum-sensing system, although other chitinases may also be present [10].

Three other identified protein entries exhibited a chitin-binding motif (carbohydrate-binding protein CV_3323, transmembrane hydrolase CV_1440, and hypothetical protein CV_1369), although their functions are still unknown. These gene products may be involved in carbohydrate metabolism or in an unrelated, unknown function; the *L. pneumophila* chitinase, for example, enables the survival of this pathogen in the lung [15].

As a bacterium that lives exposed to the environment, *C. violaceum* requires efficient stress protection systems. Two exoproteins that might be involved in oxidative stress protection were identified: superoxide dismutase (SOD) and peroxidase. SOD converts O_2_^−^, produced by the ultraviolet irradiation of water, to O_2_ and H_2_O_2_. The presence of SOD CV_0867 in the extracellular medium is necessary because O_2_^−^ cannot penetrate through membranes; thus, *C. violaceum* requires a system that is capable of detoxifying this molecule at its source [21]. Although H_2_O_2_ is able to penetrate through membranes, the presence of the peroxidase CV_3739, a member of the peroxiredoxin family, in the extracellular medium may be important for its detoxification.

Among the cytoplasmic proteins found in the extracellular milieu identified, some are related to bacteriophages (CV_0340, CV_0350, CV_0409, CV_0410, and CV_0424), which can reach the extracellular medium via holins [36]. The *C. violaceum* genome contains four prophages from different sources, designated CvP1, CvP2, CvP3, and CvP4 [13]. Proteins CV_0409, CV_0410, and CV_0424 belong to CvP2, which can display bactericidal activity [13].

Several gene products of unknown function were found in the *C. violaceum* exoproteome: CV_0223, CV_0349, CV_0408, CV_1369, CV_2893, CV_3276, CV_3475, and CV_4224. These proteins may have important roles in the adaptation of the bacterium to the diverse environments in which it can survive and the pathology it induces. Further biochemical structural characterization of these proteins is necessary for the determination of their extracellular function.

## Conclusions

The *C. violaceum* proteins identified so far on the exoproteome comprise a wide array of molecular tools, some of which with potential biotechnological applications that are fundamental to its environmental adaptation as well as an arsenal of proteins that aid in the process of host invasion and injury. These results also confirm previously described genomic data and validate the expression and localization of the gene products identified here.
